# Neurexins and their ligands at inhibitory synapses

**DOI:** 10.3389/fnsyn.2022.1087238

**Published:** 2022-12-21

**Authors:** Emma E. Boxer, Jason Aoto

**Affiliations:** Department of Pharmacology, University of Colorado Anschutz Medical Campus, Denver, CO, United States

**Keywords:** neurexins, inhibitory, GABAergic synapses, neuroligins, cerebellins, dystroglycan, neurexophilins, transsynaptic

## Abstract

Since the discovery of neurexins (Nrxns) as essential and evolutionarily conserved synaptic adhesion molecules, focus has largely centered on their functional contributions to glutamatergic synapses. Recently, significant advances to our understanding of neurexin function at GABAergic synapses have revealed that neurexins can play pleiotropic roles in regulating inhibitory synapse maintenance and function in a brain-region and synapse-specific manner. GABAergic neurons are incredibly diverse, exhibiting distinct synaptic properties, sites of innervation, neuromodulation, and plasticity. Different classes of GABAergic neurons often express distinct repertoires of Nrxn isoforms that exhibit differential alternative exon usage. Further, Nrxn ligands can be differentially expressed and can display synapse-specific localization patterns, which may contribute to the formation of a complex *trans*-synaptic molecular code that establishes the properties of inhibitory synapse function and properties of local circuitry. In this review, we will discuss how Nrxns and their ligands sculpt synaptic inhibition in a brain-region, cell-type and synapse-specific manner.

## Neurexins are synaptic organizing molecules

**FIGURE 1:**
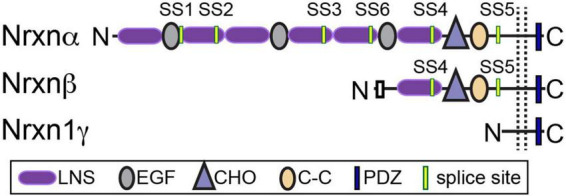
Neurexin structure. Nrxns are encoded from three genes (Nrxns 1–3). α-Nrxns (1–3) contain 6 LNS domains, 3 EGF-like-repeats, and up to 6 sites of alternative splicing. β-Nrxns (1–3) contain LNS6 and splice sites 4 and 5. γ-Nrxn (Nrxn1 only) is highly truncated. Reproduced with permission from Boxer et al. (2021).

Vertebrate neurexins (Nrxns) are essential and evolutionarily conserved presynaptic cell adhesion molecules (CAMs) that organize and critically regulate synaptic transmission of excitatory and inhibitory synapses through pleiotropic functions. Nrxns are encoded by three genes, Nrxns 1–3. Each gene encodes a longer α and shorter β isoform, and in the case of Nrxn1, a highly truncated γ isoform from independent promoters (Figure 1). α and β neurexins share transmembrane and short intracellular sequences, however, they differ in the length and complexity of their extracellular sequences. The extracellular sequences of α-Nrxns contain six laminin-neurexin-sex hormone domains (LNS1-6) with three evenly dispersed epidermal growth factor-like repeats (EGF1-3). By contrast, the extracellular sequences of β-Nrxns are far less complex: they have a unique N-terminus but share the same LNS6 domain with α-Nrxns. The Nrxn1-specific γ isoform lacks all recognized extracellular domains, except for extracellular juxtamembrane sequences. In addition to multiple Nrxn isoforms generated from a single gene, Nrxns are subject to a high degree of alternative splicing- there are 6 alternative splice sites (SS1-6) in α-Nrxns, and 2 splice-sites (SS4-5) in β-Nrxns- which together generate over a thousand possible alternative splice isoforms per neurexin (Ullrich et al., 1995; Schreiner et al., 2014; Treutlein et al., 2014). The expression of individual Nrxn isoforms and usage of alternative exons are highly differentiated among cell types and brain regions. It is proposed that at a given synapse, the repertoire and synaptic localization of individual Nrxns, along with the regulated expression profiles of their post-synaptic ligands, generate a synaptic cell adhesion combinatorial code that is proposed to coordinate the profound diversity of synaptic properties in the central nervous system (Südhof, 2017).

Since the discovery of Nrxns in 1992, the field has focused extensively on elucidating Nrxn function at excitatory synapses. Studies examining Nrxn function at inhibitory synapses, however, are only just emerging. Recent transcriptomics studies reveal that Nrxn isoform expression profiles in inhibitory cells differ drastically from those of excitatory cells. Moreover, different classes of GABAergic neurons exhibit distinct Nrxn isoform expression and/or alternative splicing profiles (Schreiner et al., 2014; Fuccillo et al., 2015; Földy et al., 2016; Nguyen T. -M. et al., 2016; Lukacsovich et al., 2019; Huntley et al., 2020). Similar to their function at excitatory synapses, Nrxns at inhibitory synapses have multiple synaptic roles that differ depending on the brain region, cell-type, synapse, and even sex of the animal studied (Table 1). Importantly, these functions are dependent on interactions with specific ligands, which also exhibit differential expression and/or localization in both the pre- and post-synaptic neuron. Interrogation of Nrxn function at inhibitory synapses is critical to understanding how these molecules play a role in the many developmental and neuropsychiatric disorders they are implicated in, including autism spectrum disorders (ASDs), developmental delay, epilepsy, schizophrenia, and substance use disorders (Südhof, 2017). Here we discuss the role of Nrxns at inhibitory synapses, including their expression properties, known functions, and known binding partners.

**TABLE 1:** Summary of inhibitory synaptic phenotypes and behavior following functional manipulations of neurexins in mice.

Gene	Cell type	Phenotype	References
Nrxn 1, 2, 3 conditional triple KO	PV-Cre	Decreased n and PV-IPSCs in mPFC (sex not specified)	Chen et al., 2017
	SST-Cre	Decreased Pr and SOM-IPSCs in mPFC (sex not specified)	Chen et al., 2017
	vGlut3-Cre	Decreased Pr, connectivity, and vGlut3-IPSCs in CA1 (male and female)	Uchigashima et al., 2020a
Nrxn3 conditional KO	Regional KO by AAV-Cre delivery	No change to IPSCs in hippocampal culture, reduction of IPSCs in olfactory bulb culture and granule cell-synapses in *ex-vivo* olfactory bulb slice	Aoto et al., 2015
	vGAT-Cre	Lethal-mice die at birth	Keum et al., 2018
	PV-Cre	Increased Pr and PV-IPSCs in female subiculum, decreased n, q, and PV-IPSCs in male subiculum Behavior: No change in observational fear	Keum et al., 2018; Boxer et al., 2021
	SST-Cre	Decreased Pr and SOM-IPSCs in ACC (males) Behavior: increased observational fear	Keum et al., 2018
Nrxn3 SS5 KO	Constitutive	Reduced IPSCs from IML synapses (putative CCKs), reduced SOM-IPSCs, no effect on PV-IPSCs in DG	Hauser et al., 2022
Nrxn 1, 3 SS4 exclusion, conditional	PV-Cre	No change to PV synapse puncta density or synapse ultrastructure in CA1 Behavior: Impaired short-term memory in novel object recognition task	Nguyen T. -M. et al., 2016
Nrxn1α KO	Constitutive	No change to CA1 mini or spontaneous IPSCs Behavior: Increased grooming, enhanced rotarod motor learning, impaired nest building, impaired pre-pulse inhibition Behavior: reduced social investigation (M and F), increased aggression (males) Decreased IPSC amplitude and inhibitory connectivity in BLA Behavior: Reduced fear expression	Etherton et al., 2009; Grayton et al., 2013; Asede et al., 2020

ACC, anterior cingulate cortex; BLA, basal lateral amygdala; CA1, cornu ammonis 1 (hippocampus); CCK, cholecystokinin; DG, dentate gyrus; IML, inner molecular layer; IPSC, inhibitory post-synaptic current; KO, knock-out; mPFC, medial prefrontal cortex; n, synapse number; Pr, release probability; PV-Cre, Parvalbumin-IRES-Cre mouse line; q, quantal size; SOM, somatostatin; SS, splice site; SST-Cre, somatostatin-IRES-Cre mouse line; vGlut3-Cre, vesicular glutamate transporter 3 Cre mouse line.

## Region and cell-type specific expression of neurexins

**FIGURE 2:**
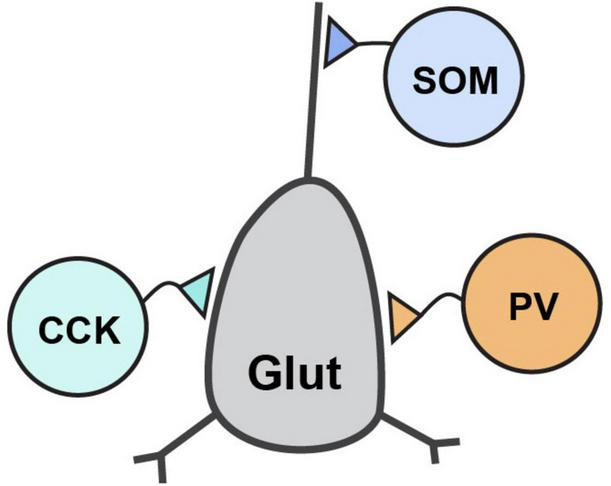
Inhibitory interneuron connectivity. Simplified diagram of the prominent forebrain inhibitory interneuron types discussed in this review and their connectivity with a post-synaptic glutamatergic neuron.

GABAergic neurons are classified by a combination of properties which includes their protein and peptide expression, receptor expression, electrophysiological properties, and morphological properties. Their defining morphological properties include features like spines, such as those found on medium spiny neurons (MSNs) of striatum, or details of their axon targeting locations which include soma, proximal and distal dendrites, and axons. The most prominent GABAergic interneurons in forebrain are the perisomatically targeting parvalbumin-expressing (PV) and cholecystokinin-expressing (CCK) neurons and the dendritically targeting somatostatin-expressing (SOM) neurons (Figure 2). These interneurons generally synapse onto glutamatergic principal neurons and have received significant attention in the CAM field due to their abundance, tractability and established roles in critically shaping neural circuit function. By contrast, GABAergic interneurons that synapse onto other GABAergic neurons, such as those expressing the vasoactive intestinal polypeptide (VIP), are also important to circuit dynamics (Guet-McCreight et al., 2020), although the function of Nrxns and their ligands at VIP synapses have received far less attention.

Parvalbumin neurons are fast-spiking interneurons that synapse onto the perisomatic regions of their post-synaptic partners. PV neurons can be further classified as basket cells, defined by their synaptic contacts with soma and proximal dendrites, or chandelier cells, defined by their synaptic contacts with the axon initial segment. Unique from SOM or CCK neurons, PV neurons express μ-opioid receptors and P/Q-type calcium channels (Freund and Katona, 2007). The other perisomatic targeting basket cell are the CCK neurons, which are regular spiking, exhibit asynchronous vesicle release, and uniquely expresses vGlut3 (in hippocampus), N-type calcium channels, and cannabinoid type 1 receptors (CB1R) (Freund, 2003; Armstrong and Soltesz, 2012). SOM neurons are dendritic-targeting interneurons and typically exhibit a low-threshold spiking phenotype, but sometimes are fast-spiking (Urban-Ciecko and Barth, 2016). While PV and SOM neurons are easily genetically accessible using Cre-driver lines, CCK neurons have been more difficult to access since CCK mRNA (but not protein) is also expressed by pyramidal neurons throughout the forebrain, and thus the CCK promoter does not restrict Cre-expression to CCK positive inhibitory neurons. Several groups have circumvented this issue using alternative approaches, such as utilizing the Dlx 5/6 promoter, active in forebrain inhibitory neurons only, to drive expression of Cre-dependent AAVs in CCK-Cre mice, (Liu et al., 2020) or similar variations of this. Other groups have taken advantage of the selective expression vGlut3, selective for a subset of hippocampal CCK interneurons (Somogyi et al., 2004), or CB1R, to access or label CCKs (Uchigashima et al., 2020a). Perhaps unsurprisingly, given the molecular, morphological and functional diversity of these GABAergic populations of neurons, Nrxn expression levels, isoform abundance and alternative exon usage also displays robust diversity.

Due to a dearth of specific Nrxn antibodies that reliably detect individual gene products, Nrxn expression has historically been interrogated at the mRNA level. The first study to examine Nrxn mRNA expression in inhibitory neurons was shortly after their discovery, wherein Ullrich et al. (1995) observed that Nrxn3 was enriched in hippocampal inhibitory neurons relative to excitatory neurons (Ullrich et al., 1995). In-depth and comprehensive analyses of Nrxn expression and alternative exon usage did not commence again in earnest until two decades later, when it was demonstrated that Nrxn isoforms differ drastically in whole tissue samples harvested from different brain regions (Aoto et al., 2013; Schreiner et al., 2014; Treutlein et al., 2014; Traunmüller et al., 2016). Tissue-specific expression was quickly superseded by the analysis of single classes of neurons, achieved *via in-situ* hybridization, single-cell quantitative reverse transcriptase (qRT)-PCR techniques (Futai et al., 2013; Fuccillo et al., 2015; Nguyen T. -M. et al., 2016; Uchigashima et al., 2019), and eventually single-cell RNA sequencing (Gokce et al., 2016; Lukacsovich et al., 2019; Boxer et al., 2021; Que et al., 2021). Analysis of single-classes of neurons revealed that Nrxn expression levels and alternative splicing exhibited remarkable cell-type-specific variability not otherwise observed in tissue samples.

### Notable isoform mRNA expression patterns in GABAergic neurons

Nrxn expression profiles of medial ganglionic eminence (MGE)-derived interneurons (such as PV and SOM) and caudal ganglionic eminence (CGE)-derived interneurons (such as CCK and VIP) (Kepecs and Fishell, 2014) are embryonically determined and remain stable into adulthood (Lukacsovich et al., 2019). While all three Nrxn genes are typically expressed in a single neuron, Nrxn2 expression is typically the lowest and is sometimes too low to confidently perform analyses on isoform expression and/or alternative exon usage (Aoto et al., 2013; Fuccillo et al., 2015; Lukacsovich et al., 2019; Boxer et al., 2021). Nrxn1 and Nrxn3 transcripts containing the alternative SS4 exon are dominantly expressed in SOM, PV, and CCK interneurons (Fuccillo et al., 2015; Nguyen T. -M. et al., 2016; Winterer et al., 2019). SS4 is located in LNS6, which is a critical binding domain for many Nrxn ligands, and the inclusion (SS4+) or exclusion (SS4-) of the SS4 insert can regulate binding to neuroligins and cerebellins, and dystroglycan (Table 2). Curiously, despite its unique expression and significance to ligand binding, studies have failed to identify the biological significance for the alternative exon usage at SS4 in mediating overall inhibitory synaptic function (discussed later) and further experiments will be required to determine its functional contribution (Nguyen T. -M. et al., 2016; Trotter et al., 2022).

**TABLE 2:** Binding requirements of Nrxns with ligands found at inhibitory synapses.

Binding partner	Neurexin isoform(s)	Neurexin binding region	Neurexin alternative splicing requirements	References
CA10/CA11	α and β	Juxtamembranous stalk	Independent of alternative splicing. *Cis*-interactions in the secretory pathway enable Nrxn trafficking	Sterky et al., 2017
Calsyntenin-3	α-specific (Pettem et al., 2013; Lu et al., 2014) α and β? (Kim et al., 2020)	N-terminal sequences of α-Nrxns (Pettem et al., 2013; Lu et al., 2014) LNS6 (Kim et al., 2020)	Independent of alternative splicing (Pettem et al., 2013) Preference for Nrxns with the SS4 insert (Kim et al., 2020)	Pettem et al., 2013; Lu et al., 2014; Um et al., 2014; Kim et al., 2020
Cerebellins/GluD1	α and β	LNS6	Requires inclusion of the SS4 insert.	Yuzaki, 2017
Dystroglycan	α and β	α-Nrxns: LNS2 β-Nrxns: LNS6	α-Nrxns: requires LNS2 lacking the SS2 insert. β-Nrxns: requires LNS6 lacking the SS4 insert.	Sugita et al., 2001
FAM19A1-4	α? and β	Juxtamembranous stalk	Independent of alternative splicing. *Cis*-interactions in the secretory pathway to regulate post-translational modification of Nrxns.	Khalaj et al., 2020
GABA_A_R	α? and β	?	Evidence of direct binding of GABA_A_Rs with Nrxns was only provided for β-Nrxns. Overexpression of α- and β-Nrxns suppresses GABAergic synaptic transmission in cultured neurons.	Zhang et al., 2010
IgSF21	Nrxn2α	LNS1	Independent of alternative splicing.	Tanabe et al., 2017
Neurexophilins	α	LNS2	Requires LNS2 lacking the SS2 insert	Missler et al., 1998
Neuroligins	α* and β	LNS6	Nlgn1 with splice-site B selectively binds β-Nrxns; Nlgn1 lacking splice-site B binds to both α- and β-Nrxns. Alternative splicing of Nrxn SS4 modulates binding. *All Nlgns possess splice site A. *Splice-site B is unique to Nlgn1. *Nlgn3 splice-site A uses two independent exons: A1 and A2.	Boucard et al., 2005; Chih et al., 2006; Comoletti et al., 2006; Koehnke et al., 2010

Except for a few noted exceptions, most ligands listed are capable of binding to all 3 Nrxns. *Represent the neurexin alternative splicing requirements. ?Refers to inconclusive.

The molecular mechanisms that regulate the selective cell-type specific usage of the alternative SS4 exon are being examined. Convincing evidence has implicated three members of the signal transduction and activation of RNA (STAR) domain family of RNA binding proteins—SAM68, SLM1, and SLM2—in regulating splicing at Nrxn SS4. SAM68, SLM1, and SLM2 possess a single central RNA-binding KH domain and their expression in neurons promotes the skipping of the SS4 exon to produce SS4- Nrxns (Chawla et al., 2009; Iijima et al., 2011, 2014; Ehrmann et al., 2013; Feracci et al., 2016; Nguyen T. -M. et al., 2016; Traunmüller et al., 2016). Of the three molecules, SLM2 appears to critically regulate the alternative splicing at SS4 of all Nrxns. SLM2 is highly expressed in hippocampal principal neurons, which results in the production of Nrxns lacking the SS4 insert. By contrast, a significant fraction of hippocampal PV neurons do not express SLM2 and express SAM68 and SLM1 at levels lower than in principal neurons, which results in the inclusion of the SS4 insert (Nguyen T. -M. et al., 2016). Curiously, while SLM2 appears to be a key determinant for splicing in PV interneurons, the majority of CA1 CCK and SOM interneurons express SLM1 or SLM2, respectively, yet exhibit SS4 exon usage similar to PV neurons (Iijima et al., 2014; Fuccillo et al., 2015; Nguyen T. -M. et al., 2016; Winterer et al., 2019). Although SAM68, SLM1, and SLM2 each have the capability to regulate SS4 usage, it will be important to further assess how each RNA-binding protein is regulated and utilized by different classes of GABAergic interneurons.

The functional relevance of the other Nrxn splice sites is not as well characterized as SS4, even at excitatory synapses. However, the other splice sites could play significant roles in inhibitory synaptic function, as their expression profiles are highly region-and cell-type specific. Notably, the alternative SS2 insert appears to be excluded (SS2-) from Nrxns 1 and 3 of both MGE and CGE-derived interneurons, but is included in Nrxn1 in pyramidal neurons (Lukacsovich et al., 2019). Accordingly, SS2- is required for binding to the inhibitory synapse Nrxn ligands dystroglycan and neurexophilins 1 and 3 (Table 2). Another notable expression pattern involves SS3, which is included in PV neurons, but excluded in CCKs in both hippocampus and cortex (Fuccillo et al., 2015; Lukacsovich et al., 2019). Interestingly, based on the possible combinations of Nrxn alternative exon usage, Nrxn3 has the largest potential for transcriptional diversity, but interestingly does not harness this potential. Instead, alternative exon usage of Nrxn3 is more restricted than predicted and displays the least possible diversity of the three neurexins at the mRNA levels (Schreiner et al., 2014).

### Protein expression and post-translational modifications

In addition to alternative splicing, post-translational modifications further add to the diversity of Nrxns. While all Nrxns are N-glycosylated, the sequences between LNS6 and the transmembrane region are subject to O-linked glycosylation (Ushkaryov et al., 1994). More recently, it was revealed that Nrxns1–3 share a conserved serine residue located immediately upstream of the conserved Cys-loop sequence that is modified by the addition of heparin sulfate (Zhang et al., 2018). Controlled by CA10 and FAM19A1-4, the heparin sulfate modification of Nrxns facilitates interactions with Nlgns and LRRTMs and has been proposed to expand the binding diversity by recruiting heparin-sulfate binding proteins to Nrxn complexes (Zhang et al., 2018; Khalaj et al., 2020; Noborn and Sterky, 2021). Additionally, heparin sulfate modifications may be regulated in an activity-dependent manner by FAM19A1-4 (Khalaj et al., 2020).

Unique to Nrxn3 is the presence of an in-frame stop codon encoded in the SS5 exon (Nrxn3 SS5+), that results in the production of Nrxn3 without transmembrane and intracellular domains (Ushkaryov and Südhof, 1993; Tabuchi and Südhof, 2002). This truncated splice isoform of Nrxn3 is post-translationally modified by the addition of a glycosylphosphatidylinositol (GPI) membrane anchor (Hauser et al., 2022). The GPI membrane anchor attaches the extracellular sequences of Nrxn3 to the plasma membrane but does not permit intracellular signaling. Furthermore, the Nrxn3 SS5 exon encodes for mRNA sequences that are subject to strong translational repression resulting in cell-type specific protein expression. Nrxn3 SS5+ mRNA is detected in pyramidal neurons and GABAergic neurons in hippocampus but its protein is only detected in inhibitory neurons (Hauser et al., 2022).

Measuring the expression and localization of Nrxn protein is no easy task due to the lack of antibodies that detect individual Nrxns (Südhof, 2017). Currently, the most reliable approach to characterize Nrxn protein requires the generation of endogenously tagged Nrxns (Anderson et al., 2015; Ribeiro et al., 2019; Trotter et al., 2019; Klatt et al., 2021; Hauser et al., 2022). However, analysis of Nrxn expression at the protein level and its targeting to specific synapses will be an important undertaking for future studies. One important but unanswered question is whether Nrxn molecules are equally distributed among synapses of a given neuron, or whether specific isoforms are selectively trafficked or retained at certain synapses depending on factors such as the identity or activity of the post-synaptic neuron (Boxer et al., 2021). Moreover, examination of the subsynaptic localization of individual Nrxn molecules, enabled by super resolution microscopy, will expand our understanding of how Nrxns interact with and control synapse organization (Trotter et al., 2019).

## Brain region, cell-type, and synapse-specific diversity of neurexin function

While Nrxn function at inhibitory synapses can be somewhat generalized in reduced systems (i.e., *in vitro* studies show that Nrxns are synaptogenic, facilitate synapse specialization, and enable neurotransmission), studying Nrxns in intact neural circuits reveals that different brain regions, presynaptic cell-types, and post-synaptic cell-types display remarkable diversity in their functional requirement and utilization of neurexins (Table 1). This functional diversity is thought to arise from the regional and cell-type specific expression of different Nrxn isoforms and alternative exon usage, as well as the differential expression of post-synaptic Nrxn binding partners.

A striking example in which Nrxn function depends on the identity of the GABAergic presynaptic cell (i.e., the class of interneuron) was found using conditional knock-out of all three Nrxns (triple KO). The triple KO of Nrxns reduced synaptic inhibition mediated by PV and SOM interneurons onto layer 5 pyramidal neurons in medial prefrontal cortex (mPFC), however, the synaptic properties impacted by the deletion of all Nrxns differed significantly. Triple KO in PV interneurons reduced the density of synapses made onto pyramidal neurons, while triple KO in SOM neurons impaired action potential-induced presynaptic calcium influx and significantly reduced presynaptic release (Chen et al., 2017). Further, in region CA1 of hippocampus, the deletion of all Nrxns from CCK interneurons using vGluT3-Cre mice reduced synaptic strength onto CA1 pyramidal neurons (Uchigashima et al., 2020a). While the triple Nrxn KO model is a useful tool to identify synapses that utilize Nrxns and begin to identify the general properties of the Nrxn family, whether these phenotypes are a result of redundant Nrxn function or primarily driven by the loss of a single Nrxn was not investigated.

In a separate study, the impact of Nrxn3 KO in SOM neurons that synapse onto pyramidal neurons in layer 2/3 of anterior cingulate cortex (ACC), a region of prefrontal cortex, was investigated. The KO of Nrxn3 from SOM neurons impaired presynaptic release probability and reduced SOM-mediated inhibitory post-synaptic current (IPSC) amplitudes at SOM-pyramidal neuron synapses (Keum et al., 2018). Importantly the single deletion of Nrxn3 in SOM neurons recapitulated the presynaptic phenotypes observed in the triple Nrxn KO (Chen et al., 2017). Together with the triple KO results, this suggests that Nrxn3 is functionally dominant at SOM synapses in prefrontal cortex. A caveat to this interpretation is that the protein repertoire of Nrxn3 isoforms and/or alternative insert usage in SOM interneurons and the post-synaptic ligand properties in pyramidal neurons may exhibit subregion or layer specificity (layer 2/3 vs. layer 5). To address this possibility, the patterns of expression and localization of endogenous Nrxns and their ligands should be assessed. Interestingly, the deletion of Nrxn3 from SOM neurons altered observational fear behavior, whereas Nrxn3 KO in PV neurons did not produce similar changes to observational fear behavior. While the synaptic phenotype of PV-Nrxn3 KO in ACC was not assessed, these behavioral findings imply that PV-mediated synaptic inhibition in ACC may not be regulated by Nrxn3 because PV interneuron activity in AAC has been shown to be critical for observational fear learning (Zhou et al., 2018).

Recent work from our lab demonstrated that in subiculum of ventral hippocampus, Nrxn3 plays an essential role in PV-mediated synaptic transmission. Similar to the triple Nrxn KO in mPFC, PV synapse numbers, post-synaptic strength, and PV-IPSC amplitudes were drastically reduced following the ablation of Nrxn3 from PV neurons (PV-Nrxn3 KO) in males (Boxer et al., 2021). However, this phenotype was curiously sex-dependent. In females, the deletion of Nrxn3 from PV interneurons enhanced the presynaptic release probability and PV-IPSC amplitudes, suggesting that in females, Nrxn3 acts to limit release probability whereas in males, it acts to stabilize synapses (Boxer et al., 2021). Furthermore, these functions were dependent on the identity of the post-synaptic cell: PV-Nrxn3 KO only affected PV synapses made onto one class of subicular pyramidal neuron, the regular spiking neurons, but had no effect at synapses made onto burst spiking neurons, indicating that in addition to sex-specific effects, Nrxn3 has synapse-specific effects in subiculum. The most parsimonious explanation for these cell-type and sex-specific differences in Nrxn3 function is differential Nrxn expression and/or alternative exon usage, however, single-cell RNA sequencing revealed that male and female PV neurons express identical Nrxn mRNA profiles (Boxer et al., 2021). It is possible that differential expression of Nrxn ligands by the post-synaptic cells play a role in the synapse and sex-specific phenotypes. Another possibility is that there are differences in Nrxn3 protein expression and/or synaptic localization that are not observed at the mRNA level. Tools to assess endogenous Nrxn3 protein expression and localization are currently limited to studying the inclusion of the Nrxn3 SS5 insert (Hauser et al., 2022). These knock-in mice, which harbor an epitope tag within SS5 of endogenous Nrxn3, revealed that Nrxn3 SS5+ exhibits differential mRNA vs. protein expression in hippocampus, discussed below.

### Manipulations of alternative splicing

Specific to Nrxn3 is a SS5 alternative exon that was recently demonstrated to regulate SOM and CCK transmission in hippocampus. Fascinatingly, the inclusion of the SS5 exon in Nrxn3 mRNA is observed in both glutamatergic and GABAergic neurons in hippocampus, but Nrxn3 SS5+ protein is completely absent from glutamatergic cells, suggesting translational repression in glutamatergic neurons (Hauser et al., 2022). In dentate gyrus, Nrxn3 SS5+ protein (visualized *via* an epitope tag inserted into endogenous Nrxn3 SS5) is present in PV, CCK, and SOM-expressing interneurons, however, it exhibits selective presynaptic enrichment only in synapses made by SOM and CCK neurons (Hauser et al., 2022). Deletion of the SS5 exon resulted in the selective impairment of SOM-and CCK-mediated transmission without altering PV-mediated transmission or excitatory transmission. This study suggested that Nrxn3 SS5 controls CCK synapse formation or stabilization as the authors observed a slight but significant reduction in the number of synapses formed by Nrxn3 SS5 KO CCK neurons in dentate gyrus, but the synaptic property controlled by Nrxn3 SS5 at SOM synapses was not identified. However, cellular binding assays and elegant proteomics on novel Nrxn3 SS5 HA-tagged mice revealed neurexophilin 1 (Nxph1) and Fam19a2, which bind to Nrxns in *cis*, as binding partners that may mediate these synaptic functions (Hauser et al., 2022).

Nrxn SS4 is a critical binding site for many inhibitory Nrxn ligands such as neuroligins, dystroglycan, and cerebellins, and governs properties at excitatory synapses in hippocampus. Specifically, Nrxn1 SS4+ controls NMDAR currents whereas Nrxn3 SS4- is required for AMPAR-mediated synaptic transmission (Aoto et al., 2013; Dai et al., 2019, 2021). Inhibitory neurons throughout forebrain predominantly express Nrxn1 and Nrxn3 mRNAs that include the SS4 exon (Fuccillo et al., 2015; Nguyen T. -M. et al., 2016; Lukacsovich et al., 2019; Boxer et al., 2021; Que et al., 2021). Together, these observations have led to the prediction that SS4 must be critical for inhibitory synaptic function. Surprisingly, however, the selective genetic manipulation of SS4 in Nrxn1 and Nrxn3 in PV interneurons had only modest effects on behavior and had no effect on PV synapse numbers (Nguyen T. -M. et al., 2016). Further, a recent study indicated that SS4 of Nrxn3 may not be necessary for inhibitory function in olfactory bulb or mPFC, despite Nrxn3 being predominantly expressed as the SS4+ variant in inhibitory neurons in these regions as well. Interestingly, inhibition in olfactory bulb and mPFC may depend on alternative exon usage at both SS2 and SS4, which in concert control inhibitory synaptic function *via* interactions with dystroglycan (Trotter et al., 2022).

### Conclusion from functional studies of neurexins

The studies reviewed above demonstrate that Nrxns play pivotal roles in regulating multiple aspects of inhibitory synaptic transmission that is dependent on the brain region, GABAergic cell-type and synapse studied. At PV- and SOM-expressing GABAergic synapses made onto layer 5 pyramidal neurons in mPFC, the ablation of all Nrxns resulted in a reduction in synapse density and presynaptic release probability, respectively (Chen et al., 2017). At CCK synapses in hippocampal slice cultures, Nrxn triple KO impaired release probability (Uchigashima et al., 2020a). When studying the contributions of individual Nrxns to synaptic inhibition, the field has focused primarily on interrogating the functional properties of GABAergic synapses controlled by Nrxn3. Genetic manipulation of Nrxn3 in Layer 2/3 SOM neurons in ACC resulted in reduced presynaptic release probability, which recapitulated the triple Nrxn KO phenotype (Chen et al., 2017; Keum et al., 2018). In the dentate gyrus of hippocampus, manipulation of the Nrxn3 SS5 insert revealed a role for Nrxn3 SS5 in controlling the strength of CCK- and SOM-expressing synapses onto pyramidal neurons (Hauser et al., 2022). In the subiculum of hippocampus, we found that Nrxn3 plays a critical sex- and synapse-specific role in regulating inhibition exclusively at synapses made by PV interneurons onto regular spiking neurons. In males, Nrxn3 is required to promote synapse maintenance and post-synaptic strength. In females, Nrxn3 suppresses presynaptic release probability (Boxer et al., 2021). Together these findings raise two important questions: (1) How can Nrxn3 display such striking functional heterogeneity? and (2) Do Nrxns other than Nrxn3 also play critical roles in synaptic inhibition?

The function of Nrxns can be influenced by alternative exon usage (Aoto et al., 2013; Dai et al., 2019, 2021), synaptic localization (Hauser et al., 2022), and ligand availability (Uchigashima et al., 2020a). Nrxn alternative splicing varies among classes of hippocampal interneurons, which may result in the formation of different *trans*-synaptic complexes to differentially sculpt synaptic transmission onto principal neurons (Fuccillo et al., 2015; Nguyen T. -M. et al., 2016; Uchigashima et al., 2020a). As discussed above, studies have failed to find a critical role for the SS4 insert in Nrxn-dependent synaptic inhibition, however, SS2 and SS3 exhibit differential exon usage in distinct classes of GABAergic neurons and SS2 is a binding site for some inhibitory ligands (discussed below) (Fuccillo et al., 2015; Südhof, 2017; Lukacsovich et al., 2019). Although the precise mechanisms that influence the trafficking of Nrxns to presynaptic terminals are poorly understood, Hauser et al. (2022) revealed that the inclusion of the SS5 insert in Nrxn3 selectively localizes Nrxn3 to the axon terminals of CCK and SOM, but not PV interneurons. As expected, deletion of the SS5 insert selectively impacted GABAergic synaptic transmission at synapses made by CCK and SOM interneurons but not PV interneurons. These findings emphasize the need to not only understand Nrxn mRNA expression profiles but also determine the subcellular properties of Nrxns. To circumvent the lack of reliable Nrxn antibodies, groups have developed elegant epitope-tagged Nrxn mice to profile full-length Nrxn1 (Ribeiro et al., 2019; Trotter et al., 2019), all β-Nrxns (Anderson et al., 2015; Klatt et al., 2021) and specifically Nrxn3 with an insert at SS5 (Hauser et al., 2022). However, given that Nrxn3 plays an important role in synaptic inhibition, the development of epitope-tagged mice that permit the assessment of full-length Nrxn3 is necessary. Moreover, as discussed below, Nrxn ligands may not be uniformly expressed in all pyramidal neurons and may not be uniformly distributed to all inhibitory synapses; instead, some ligands are differentially expressed in a brain region-specific manner and can be preferentially enriched post-synaptically at subsets of inhibitory synapses (Lukacsovich et al., 2019; Uchigashima et al., 2020a). Another potential explanation for why Nrxn3 exhibits pleiotropic function at different inhibitory synapses is that signaling mediated by the cytoplasmic sequences of Nrxn3 may be differentially utilized in distinct classes of GABAergic neurons.

Finally, along with Nrxn3, Nrxn1 is highly expressed in most GABAergic neurons in cortex and hippocampus (Nguyen T. -M. et al., 2016; Lukacsovich et al., 2019; Uchigashima et al., 2020a; Boxer et al., 2021; Que et al., 2021) yet the impact of Nrxn1 on synaptic inhibition is poorly characterized. Overexpression experiments hint that Nrxn1 and Nrxn3 may be differentially used at inhibitory synapses: at CCK GABAergic synapses onto CA1 pyramidal neurons in organotypic slices, overexpression of Nrxn1, but not Nrxn3, was capable of partially rescuing the Nrxn triple KO phenotype (Uchigashima et al., 2020a). A systematic assessment of individual neurexin function at GABAergic synapses may provide new insight into how individual Nrxns contribute to altered inhibition observed in neuropsychiatric, neurodevelopmental and substance use disorders.

## Activity dependent neurexin expression

Exciting new studies indicate that experience and behavior can modify Nrxn mRNA levels and alternative exon usage in a region, cell-type, and sex-specific manner. Changes in Nrxn mRNA expression and alternative splicing are observed following *in-vivo* drug administration in nucleus accumbens (Fuccillo et al., 2015) and globus pallidus (Kelai et al., 2008). Modifications to the inclusion of SS4 are commonly observed in multiple brain regions following several experiences, including fear conditioning (Rozic et al., 2011; Ding et al., 2017), stress (Freire-Cobo and Wang, 2020), and exercise (Innocenzi et al., 2021). In studies that distinguished multiple cell-types or brain regions, Nrxn expression profiles displayed cell-type and/or region-specific changes, suggesting that Nrxn expression profiles change in accordance with circuit-specific plasticity. This is supported by a study that found that following fear conditioning, only hippocampal neurons that are selectively activated during fear learning recall, i.e., the neuronal memory engram, exhibited changes in Nrxn splicing (Ding et al., 2017).

In contrast to glutamatergic neurons, where the SS4 insert for Nrxn1 and 3 is well-established to regulate glutamatergic LTP in hippocampus (Aoto et al., 2013; Dai et al., 2019, 2021) a functional role for any Nrxn in mediating inhibitory synaptic plasticity has not been explored. Long term plasticity differentially manifests at PV, SOM, and CCK synapses in cortex and hippocampus to dynamically influence glutamatergic plasticity or maintain excitatory/inhibitory (E/I) balance (Chiu et al., 2019; Udakis et al., 2020). The synaptic properties altered in examples of inhibitory synaptic plasticity, such as presynaptic release probability, synapse density, and post-synaptic strength, are properties controlled by neurexins at PV, SOM, and CCK synapses (discussed below), suggesting that neurexins may be necessary for mediating these changes.

## Function of neurexin binding partners at inhibitory synapses

Shortly following the discovery of neurexins as the neuronal surface receptor for the black widow venom, α-latrotoxin (Ushkaryov et al., 1992), The Südhof laboratory began identifying pre- and post-synaptic binding partners of the neurexin family. Intracellularly, neurexins directly bind to synaptotagmin, CASK, syntenin, and Mints, *via* their conserved c-termini (Hata et al., 1993, 1996; Biederer and Südhof, 2000) thus linking them to synaptic vesicles and the synaptic vesicle release complex. The first post-synaptic binding partners to be identified were neurexophilin and neuroligin 1 (Ichtchenko et al., 1995; Petrenko et al., 1996). At this time, α vs. β neurexins and different alternative splice isoforms were already being recognized for their differential extracellular binding preferences. Surprisingly, the identification of cytoplasmic binding partners for Nrxns has been relatively stagnant compared to the pace at which extracellular binding partners have been identified. The development of epitope-tagged Nrxn mice may enable the identification new cytoplasmic binding partners to expand our understanding of the intracellular signaling pathways downstream of individual Nrxns.

**FIGURE 3:**
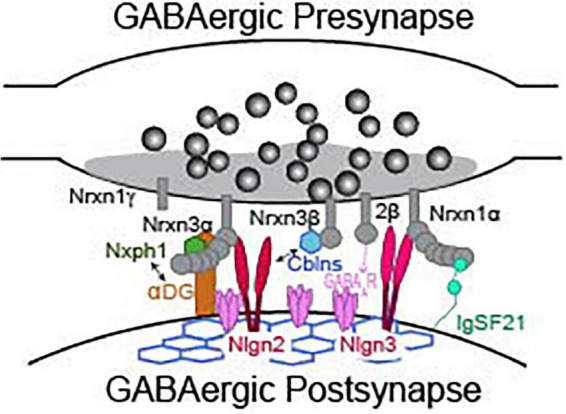
Presynaptic neurexins and their binding partners at a GABAergic synapse. Not representative of any particular type of inhibitory synapse. See Table 2 for details about binding requirements.

While direct analyses of Nrxns are the most straightforward way to interrogate their function, we can gain profound insight to their synaptic function by reviewing functional studies of Nrxn binding partners. Studies of known Nrxn binding partners at inhibitory synapses, such as neuroligins, cerebellins, dystroglycan, neurexophilins, calsyntenin-3, carbonic anhydrase related proteins 10 and 11 (CA10/11), FAM19A1-4, GABA_A_R, and immunoglobulin superfamily member 21 (IgSF21) (Figure 3), are far less numerous than studies of Nrxns and their ligands at excitatory synapses. Due to advances in the sensitivity of biochemical and proteomic assays, new binding partners for Nrxns are still being identified. Importantly, the isoform identity (α vs. β) and alternative splicing at splice sites 2 and 4 of Nrxns play significant roles in defining the binding affinity with most known ligands (Table 2). The modulation of ligand binding by Nrxn isoforms and alternative splicing supports the notion that Nrxn complexes participate in a “molecular code” that is instructive for synapse maintenance and function. Notable exceptions are Nrxn binding with GABA_A_Rs, calsyntenin-3, CA10/11, and FAM19a1-4 which appear to occur independently of these structural variables (Zhang et al., 2010; Pettem et al., 2013; Sterky et al., 2017; Khalaj et al., 2020; Liu et al., 2022). CA10/11 and FAM19a1-4 bind to Nrxns in *cis* (Table 2) and regulate surface trafficking and post-translational modifications of Nrxns (Sterky et al., 2017; Khalaj et al., 2020). While these newly identified ligands have been rigorously interrogated biochemically, an understanding of their contribution to inhibitory synaptic transmission is currently limited and likely indirect, and will not be reviewed here. Although Nrxns appear to be the preferred presynaptic binding partners of these newly identified ligands, it is, however, important to keep in mind that these post-synaptic molecules may have binding partners beyond just Nrxns and thus may participate in Nrxn-independent functions. Thus, care must be taken when inferring Nrxn function from these studies, and conclusions should only be drawn when Nrxn function is directly experimentally tested.

### Neuroligin 2

There are four Nlgn genes, Nlgns 1–4, but Nlgns 1–3 are most often studied in rodents, as Nlgn4 function is not conserved between rodents and humans (Nlgn4 was originally linked to inhibitory synapses in mice but regulates excitatory synapses in humans). Nlgns were quickly appreciated for their powerful synaptogenic properties- when expressed in non-neuronal cells or coated onto beads, these molecules rapidly induced the recruitment of presynaptic specializations (Scheiffele et al., 2000; Graf et al., 2004; Nam and Chen, 2005; Südhof, 2017). However, knockout studies revealed no differences in synapse formation, but significant differences in synapse function (Varoqueaux et al., 2006; Chubykin et al., 2007; Liang et al., 2015; Zhang et al., 2015; Chanda et al., 2017). Nlgn1 is exclusive to excitatory synapses and will not be discussed in detail here. Relevant to inhibitory synapses are Nlgn2 and Nlgn3. Nlgn2 is localized to GABAergic (Graf et al., 2004; Varoqueaux et al., 2004), dopaminergic, and cholinergic synapses (Takács et al., 2013; Uchigashima et al., 2016). Its localization to dopaminergic and cholinergic synapses may potentially be because they co-release GABA (Südhof, 2017). Nrxns are the only known presynaptic receptor for Nlgn2 and engage in *trans*-synaptic interactions to control synaptic function. Trans-synaptic interactions between Nrxns and Nlgn2 are regulated by alternative splicing at splice-site 4 of Nrxns and by alternative splicing of splice-site A of Nlgn2 (Ichtchenko et al., 1996; Chih et al., 2006; Koehnke et al., 2010). Splice-site A is conserved in all Nlgns. Unlike Nlgn1, Nlgn2 is competent to interact with both α- and β-Nrxn isoforms. Nlgn1 uniquely possesses a second splice-site (splice-site B) that dictates binding to α-Nrxns, which is absent in Nlgn2 (Chih et al., 2005, 2006; Patrizi et al., 2008; Nguyen Q. -A. et al., 2016).

Nlgn2 is recruited to inhibitory synapses independent of GABA_A_R activity and then recruits gephyrin, the central scaffolding molecule at inhibitory synapses, and activates collybistin to recruit and organize inhibitory post-synaptic proteins, including GABA_A_Rs (Graf et al., 2004; Patrizi et al., 2008; Poulopoulos et al., 2009; Ali et al., 2020). Knockout studies indicate that Nlgn2 is required both during development to assemble the post-synaptic components of inhibitory synapses, and throughout adulthood for continuous synapse maintenance (Liang et al., 2015; Troyano-Rodriguez et al., 2019). Across brain regions, imaging studies consistently reveal that Nlgn2 KO impairs post-synaptic but not presynaptic morphology, indicating that Nlgn2 does not control presynaptic organization (Ali et al., 2020). Interestingly, this is in contrast to Nlgns 1 and 3, which participate in the regulation of both post- and pre-synaptic properties (Uchigashima et al., 2021).

Curiously, while Nlgn2 is ubiquitous at inhibitory synapses, Nlgn2 KO primarily affects perisomatic synapses. In hippocampus and basolateral amygdala, Nlgn2 KO selectively impacts GABA_A_Rs and gephyrin at perisomatic synapses (Poulopoulos et al., 2009; Jedlicka et al., 2011; Babaev et al., 2016); in cortex, Nlgn2 KO mice exhibit reduced inhibition at PV, but not SOM synapses (Gibson et al., 2009). However, note that in addition to impairment at PV synapses, Horn et al. did find that Nlgn2 knock-down in organotypic hippocampal slices impaired SOM-interneuron transmission (Horn and Nicoll, 2018). Nlgn2 KO from Purkinje cells in cerebellum reduced total inhibitory synaptic strength but the relative impact of the KO on perisomatic-targeting basket cells and distal-targeting stellate cells was not tested (Zhang et al., 2015). Surprisingly, despite the fact that CCKs are the other primary perisomatic targeting interneuron in the forebrain, the requirement for Nlgn2 in CCK inhibitory transmission has not been directly assessed. Moreover, Nlgn2 localizes with dystroglycan at somatic synapses, further indicating Nlgn2 may play a yet tested role at CCK synapses (see below for further discussion in section “Dystroglycan”).

MAM-domain containing GPI anchor proteins, MDGAs, are post-synaptic membrane proteins that bind to Nlgn2 with high affinity and compete with Nrxns for Nlgn binding. Overexpression and knock-down studies demonstrate that MGDAs limit the abundance of inhibitory, but not excitatory synapses, in a Nlgn2-dependent manner, and thus MGDAs inhibit Nlgn2’s ability to function at inhibitory synapses (Lee et al., 2013). MGDAs have an important role in neuronal migration and neurogenesis early in development, and MGDA2 KO is lethal.

### Neuroligin 3

Of the four Nlgns, Nlgn3 is perhaps the most extensively studied, despite its genetic KO having modest impacts on synaptic function (Südhof, 2017). Nlgn3 is found at both excitatory and inhibitory synapses. Its localization to inhibitory synapses appears to rely on extracellular interactions in *cis* with Nlgn2 (Nguyen Q. -A. et al., 2016). Functional studies examining Nlgn3 have utilized multiple approaches including ASD-associated Nlgn3 R704C or R451C KI mice, Nlgn3 KO models or Nlgn3 KD/Nlgn3 overexpression *via* biolistic transfection or viral transduction (see Uchigashima et al., 2021 for in-depth review of these models). These manipulations, extensively studied in hippocampus and somatosensory cortex, have identified inhibitory synaptic properties controlled by Nlgn3 that are distinct from Nlgn2 (Földy et al., 2013; Speed et al., 2015; Horn and Nicoll, 2018).

The Nlgn3 R451C mutation impairs surface trafficking when expressed in non-neuronal cells (Comoletti, 2004; Chubykin et al., 2005) and reduces the ability of Nlgn3 to traffic to the synapse in neurons (Chih et al., 2004; Tabuchi et al., 2007) and produces brain-region specific changes in inhibition (Tabuchi et al., 2007; Etherton et al., 2011). In somatosensory cortex of Nlgn3 R451C mice, inhibitory synaptic transmission is increased without discernable differences in excitatory synaptic transmission (Tabuchi et al., 2007). A subsequent study revealed that the increased inhibition in somatosensory cortex stemmed from decreased endocannabinoid (eCB)-mediated suppression of IPSCs and that PV and SOM-mediated IPSCs were unaltered (Speed et al., 2015), indicating that the increased inhibition could be driven by increased release from eCB sensitive CCK interneurons. By contrast, in CA1 of hippocampus, excitatory but not inhibitory synaptic transmission was increased in the R451C mutant mice (Etherton et al., 2011). Intriguingly, although total inhibition in CA1 was unaltered, further studies revealed synapse-specific R451C-dependent phenotypes. Synaptically connected paired recordings revealed that CCK-mediated inhibition was increased due to impaired tonic, but not phasic eCB signaling (Földy et al., 2013). Additionally, the R451C mutation decreased PV-mediated inhibition onto CA1 pyramidal neurons (Földy et al., 2013). The contrasting effects of the R451C mutation at synapses made by CCK and PV neurons on pyramidal neurons may begin to explain why changes in total inhibition was not observed in CA1. Further adding to the complexity of Nlgn3 R451C function, inhibitory synaptic transmission is reduced in basolateral amygdala (Hosie et al., 2018).

While eCB dependent phenotypes observed at CCK-positive synapses onto CA1 pyramidal neurons could be hypothesized to arise from impaired communication of Nlgn3 R451C with Nrxns, which have been shown to *trans*-synaptically mediate eCB signaling at excitatory synapses (Anderson et al., 2015). These effects may instead be mediated *via* impaired Nlgn3 binding to protein tyrosine phosphatase delta (PTPδ). PTPδ is a member of the type-II receptor protein tyrosine phosphatases, which, like Nrxns, are important presynaptic hubs for synaptic organization (Takahashi and Craig, 2013; Yoshida et al., 2021). Yoshida et al. recently found that PTPδ competes with Nrxn1 binding to Nlgn3. The selective disruption of PTPδ-Nlgn3 binding recapitulated many of the biochemical, physiological, and behavioral endophenotypes specifically observed in R451C KI mice. By contrast, disruption of Nlgn3-Nrxn binding did not resemble R451C KI mice, suggesting that the R451C KI phenotype is largely caused by impaired PTPδ binding. In fact, impairing Nlgn-Nrxn binding actually improved some of the social behavioral deficits that are observed in the R451C mice. These unexpected findings underscore how critical the diversity and precision of CAM *trans*-synaptic signaling is to the shaping neural circuits and behavior (Yoshida et al., 2021).

Interestingly, most of the phenotypes observed in cortex and hippocampus of Nlgn3 R451C KI mice are not phenocopied in Nlgn3 KO mice (Tabuchi et al., 2007; Etherton et al., 2009). However, the increased inhibition specifically at CCK synapses in CA1 of R451C mice is also present in Nlgn3 KO mice (Földy et al., 2013). By contrast, sparse knock-down (KD) of Nlgn3 in CA1 pyramidal neurons of hippocampal slice cultures caused a reduction of CCK IPSC amplitudes (Uchigashima et al., 2020a). The disparate Nlgn3 phenotypes observed in R451C KI mice vs. constitutive KO mice vs. sparse KD in slices could reflect differences in approach and/or requirement of neurexins. While the R451C mutation selectively disrupts PTPδ binding in cortex, Nlgn3 KO prevents both PTPδ and Nrxn binding. Differences in constitutive Nlgn3 KO vs. sparse Nlgn3 KD *in-vitro* are also distinct manipulations that could explain the different phenotypes. Nlgn3 KO could produce yet-unidentified developmental or compensatory effects that drive the observed functional differences. Similarly, shRNA knockdown or overexpression of Nrxns and their ligands may produce unintended consequences to perisomatic inhibition. Importantly, regardless of the differential approaches, these studies show that Nlgn3 signaling is critical at CCK synapses.

Finally, overexpression of certain Nlgn3 isoforms in CA1 pyramidal neurons can either enhance or suppress IPSCs. Splice-site A of Nlgn3 consists of two exons: A1 and A2, whose inclusion or exclusion yields four Nlgn3 splice isoforms which differ in their extracellular sequences (Nlgn3Δ, +A1, +A2, and +A1A2). Overexpression of Nlgn3 lacking splice-site A (Nlgn3Δ) or only including splice-site A2 enhances IPSCs, whereas inclusion of just A1 or both A1A2 decreases IPSCs (Uchigashima et al., 2020b). Furthermore, Nlgn3Δ and A1 mediate CCK synapses and Nlgn3 A2 mediates SOM synapses, whereas Nlgn3A1A2 may function at excitatory synapses (Horn and Nicoll, 2018; Uchigashima et al., 2020a,b).

Beyond cortex and hippocampus, Nlgn3 also mediates cerebellar and striatal inhibitory synapses in a cell-type and input-specific manner. In cerebellum, Nlgn3 is localized at a subset of inhibitory synapses, including at molecular layer interneuron-Purkinje cell synapses and at synapses in the inner granular layer (Baudouin et al., 2012; Lai et al., 2021). In nucleus accumbens, Nlgn3 expression is highly enriched in D1R compared to D2R MSNs, and accordingly, Nlgn3 KO impairs mIPSCs in D1R, but not D2R MSNs (Rothwell et al., 2014).

### Cerebellins and GluDs

Only α and β neurexins with an insert at splice site 4 (SS4+) bind to secreted cerebellins (Cblns)1, 2, or 4 and form a tripartite complex with post-synaptic ionotropic glutamate delta receptors, GluD1 or GluD2 (Yuzaki, 2017). Cerebellins are hexameric glycoproteins of the C1q and tumor necrosis factor superfamily (Kishore et al., 2004) that, depending on the synapse in question, are secreted pre- or post-synaptically. Tripartite Nrxn-Cbln-GluD complex formation regulates *trans*-synaptic organization, synapse maintenance and synaptic plasticity independent of ion flux through their pore (Fossati et al., 2019; Dai et al., 2021). The functional relevance of these tripartite signaling complexes have historically been studied at excitatory synapses in cerebellum, and more recently at excitatory synapses in subiculum, however, their roles at inhibitory synapses have only recently garnered interest.

The expression patterns of Cbln1-4 are developmentally regulated and vary dramatically by brain region and cell type. Cbln1, 2, and 4 are expressed abundantly throughout the brain while Cbln3 expression is largely restricted to neurons in cerebellum (Pang et al., 2000; Miura et al., 2006; Seigneur and Südhof, 2017). Triple knock-out of Cbln1, 2, and 4 causes reductions in excitatory synapse density in old (6 month) but not young (1–2 month) adult mice at select synapses, indicating Cblns are not required for initial synapse formation, but are utilized dynamically through development to maintain established synapses (Seigneur and Südhof, 2018). In inhibitory neurons, Cblns are differentially expressed in a cell-type-specific manner. For example, in hippocampus, PV inhibitory neurons highly express Cbln4 but not Cbln2 (Nguyen Q. -A. et al., 2016; Seigneur and Südhof, 2017; Boxer et al., 2021). Curiously, in cortex, PV neurons do not express Cblns (Lukacsovich et al., 2019). Note that this does not exclude their involvement at PV inhibitory synapses in these regions, as Cblns could still be localized to synapses by secretion from the post-synaptic glutamatergic neuron. The interrogation of Cbln signaling at inhibitory synapses is at its infancy and continued study to characterize the possible synaptic functions mediated by Cblns and their binding partners is necessary.

Studies of GluD1 and GluD2 have focused on their functional roles at excitatory synapses in cerebellum and hippocampus (Konno et al., 2014; Hepp et al., 2015; Yuzaki, 2017; Dai et al., 2021). In addition to their roles at excitatory synapses, Nrxn signaling *via* Cbln/GluD complexes are prime binding candidates to regulate inhibitory synapse function because Nrxns predominantly express the alternative SS4 exon, which is required to bind to Cblns to generate a synaptic Nrxn-Cbln-GluD tripartite complex. Indeed, several studies have shown that GluD1 and Cblns are critical for inhibitory synapse formation and specification. GluD1 expressed in HEK cells primarily induced inhibitory presynaptic differentiation when co-cultured with cortical neurons when Cbln1 or 2 were added to the media (Yasumura et al., 2012). Similarly, inhibitory pre-synapses were also formed if HEK cells were co-cultured with entorhinal cortex neurons, which intrinsically express Cbln1 (Ryu et al., 2012). In another study, GluD1 was observed at 50% of inhibitory synapses on pyramidal neurons in layer 2/3 of somatosensory cortex, and GluD1 knockdown decreased the number of inhibitory synapses formed on the dendrites of pyramidal neurons. Consistent with the morphological phenotype, GluD1 knockdown resulted in a significant reduction in mIPSC frequency but not amplitude. The regulation of synapse density by GluD1 in somatosensory cortex is dependent on binding to Cbln4, which is expressed in presynaptic somatostatin interneurons (Fossati et al., 2019). Whether neurexins are required for GluD1/Cbln4 signaling at SOM synapses in somatosensory cortex remains untested. Finally, in nucleus accumbens core of ventral striatum, GluD1 loss led to reduced mIPSC amplitude and frequency, density of inhibitory terminals, and inhibitory release probability, suggesting pre- and post-synaptic effects on synaptic transmission (Gawande et al., 2021). Loss of GluD1 in dorsal striatum, however, produced excitatory but not inhibitory deficits, indicating region-specific localization and function of GluD1.

### Dystroglycan

Dystroglycan is a Nrxn ligand found exclusively at inhibitory synapses (Lévi et al., 2002). The dystroglycan (DG) gene, Dag1, encodes a single polypeptide that is cleaved into α-DG and β-DG (Ibraghimov-Beskrovnaya et al., 1992). α-DG binds to the LNS2 region of α-Nrxns and to the LNS6 region of α and β-Nrxns (Sugita et al., 2001). Importantly, α-DG binding is dependent on alternative splicing: α-DG only binds to Nrxns that lack inserts at splice-site 2 (located in LNS2) and/or splice-site 4 (located in LNS6). Thus, α-DG competes with neurexophilin for α-Nrxn binding at the LNS2 site, and competes with Nlgns for α- and β-Nrxn binding at the LNS6 site (Missler et al., 1998; Reissner et al., 2014). Both α- and β-DG associate with the post-synaptic dystrophin-glycoprotein complex (DGC), which also contains dystrophin, dystrobrevin, and sarcoglycans. The DGC is found exclusively at perisomatic synapses in neocortex (Lidov et al., 1990) but can be found at both perisomatic and dendritic inhibitory synapses in purkinje cells of cerebellum (Sassoè-Pognetto et al., 2011; Briatore et al., 2020).

Several studies have emphasized a critical role for dystroglycan in the assembly and maintenance of CCK synapses in hippocampus and cortex: α-DG is not found opposite PV basket cell terminals, and deletion of Dag1 from pyramidal neurons selectively impairs CCK synapse assembly and maintenance as well as CCK neuron survival, without affecting PV or SOM interneurons in these regions (Früh et al., 2016; Miller and Wright, 2020, 2021). The role that Nrxn signaling plays in dystroglycan-mediated CCK maintenance is unclear and should be tested because in addition to Nrxns, dystroglycan also binds laminin and other LNS-domain containing proteins. Interestingly, Nlgn2 binds to the dystrophin-glycoprotein complex *via* S-SCAM, yet a functional role for Nlgn2 at CCK synapses has not been explored (Sumita et al., 2007; Patrizi et al., 2008). Furthermore, Nlgn3 and 4 may also interact with the DGC *via* syntrophin (Yamakawa et al., 2007). Multiple studies suggest that dystroglycan helps to recruit Nlgn2 to DGC+ inhibitory synapses, and that dystroglycan and Nlgn2 may then recruit GABAARs (Brünig et al., 2002; Panzanelli et al., 2017; Briatore et al., 2020). Together these studies suggest that the dystrophin-glycoprotein complex and the α-DG adhesion molecule are critical organizers of the CCK inhibitory postsynapse, recruiting Nlgns 2, 3, 4, and GABA_A_Rs.

α-DG is required for homeostatic synaptic plasticity by increasing GABAergic currents following prolonged elevation of neuronal activity in hippocampus (Pribiag et al., 2014). Furthermore, chronic stress downregulates α-DG expression in ventral hippocampus (Xie et al., 2022). *In-vivo* administration of agrin, a dystroglycan ligand, rescues the stress-induced behavioral impairments. A role for neurexins in these intriguing phenotypes was not tested, but may be worth investigating in future studies, as a role for Nrxns in mediating plasticity at inhibitory synapses remains untested.

### Neurexophilins

Neurexophilins are a family of small, secreted glycoproteins that are encoded by four genes in mouse (Nxphs1–4). Nxph genes are highly conserved in vertebrates but are absent in invertebrates (Wilson et al., 2019). Intriguingly, the Nxphs do not share sequence homology with any known Nrxn ligand. Nxph mRNAs exhibit differential expression patterns in brain: Nrxph1 is enriched in hippocampal interneurons, Nrxph3 is enriched in excitatory cortical neurons and Nxph4 in inhibitory hindbrain neurons (Petrenko et al., 1996; Földy et al., 2016; Tasic et al., 2016; Saunders et al., 2018; Zeisel et al., 2018). Nxph1 is arguably the most well-studied of the Nxphs and elegant genetic experiments revealed that Nxph1 signaling through α-Nrxns is required to recruit and stabilize metabotropic GABA_B_Rs and GABA_A_Rs at inhibitory synapses in thalamus (Born et al., 2014). Nxph1 was recently identified as a prominent binding partner of Nrxn3 SS5+ expressed in inhibitory neurons in hippocampus (Hauser et al., 2022). Recently, Nxph4, expressed in presynaptic Golgi cells in the cerebellum, was revealed to control GABAergic transmission at Golgi cell—granule cell synapses. Nxph4 KO mice displayed reduced inhibitory synapses as revealed by imaging and electrophysiology experiments (Meng et al., 2019). Further investigation is required to determine if Nxphs control synapse-specific properties in other brain regions in a manner similar to the Nlgns and Cblns, and to continue to elucidate the unique role of α-Nrxns at inhibitory synapses.

### Calsyntenins

Calsyntenins are post-synaptic transmembrane proteins encoded by three evolutionarily conserved genes (Cstn1-3). The extracellular sequences of Cstns contain two cadherin domains and an LNS domain (Vogt et al., 2001; Araki et al., 2003). Although there is conflicting evidence, Cstn3 was identified as an α-Nrxn specific ligand in an unbiased screen designed to identify synaptogenic proteins (Pettem et al., 2013). When expressed in non-neuronal cells and co-cultured with primary hippocampal neurons, Cstn3, but not Cstn1 or Cstn2, robustly recruited excitatory and inhibitory presynaptic specializations (Pettem et al., 2013; Um et al., 2014). However, the manipulation of Cstn3 in neurons has produced differing phenotypes. Genetic deletion of Cstn3 reduced the number of excitatory and inhibitory synapses and reduced the frequency of mEPSCs and mIPSCs in acute hippocampal slices (Pettem et al., 2013). In contrast to these findings, no changes in inhibitory synapse density were observed after shRNA-mediated knockdown of Cstn3 in cultured hippocampal neurons or in hippocampal slices from an independently generated Cstn3 KO mouse (Um et al., 2014; Kim et al., 2020). In support of the notion that Cstn3 functions at inhibitory synapses, Südhof et al. recently studied the impact of CRISPR knockdown of Cstn3 in cerebellar Purkinje neurons and found that the loss of Cstn3 reduced the density of inhibitory synapses formed by basket and stellate cells and significantly reduced mIPSC frequency and evoked IPSC amplitudes (Liu et al., 2022).

There are conflicting reports regarding the binding of Cstn3 with Nrxns. A series of elegant cell binding and biochemical and electron tomography assays identified direct interactions between Cstn3 and α-Nrxns, but not β-Nrxns, which occurred independent of SS4 (Pettem et al., 2013; Lu et al., 2014). This interaction likely requires the LNS domain of Cstn3 with extracellular sequences unique to α-Nrxns (Lu et al., 2014). However, a separate study used mass spectrometry and biochemical and cellular assays and found that Cstn3 binds to both α-Nrxns and β-Nrxns in a SS4-dependent manner (Kim et al., 2020). To add further complexity, another study failed to identify direct interactions between Cstn3 and Nrxns (Um et al., 2014). While the role for Cstn3 at inhibitory synapses is promising, it will be critical to ascertain whether the KO phenotypes reported by Pettem et al. (2013) and Liu et al. (2022) are due to the disruption of Nrxn-Cstn3 interactions or through the disruption of Cstn3 interactions with other binding partners. Additionally, it will be important to address the confusion about the Nrxn isoforms and alternative SS4 usage required for Cstn3 binding.

### GABA_A_R

In addition to interacting with post-synaptic ligands to indirectly regulate the post-synaptic strength of inhibitory synapses, Nrxns directly interact with post-synaptic GABA_A_Rs to impair the maturation of inhibitory synapses (Zhang et al., 2010). This direct interaction was demonstrated by multiple biochemical assays including affinity chromatography, reciprocal precipitation with immobilized protein and by surface-plasmon resonance. In cultured neurons, overexpression of neurexins mediated by lentiviral transduction or transient transfection reduced the strength of synaptic inhibition. Although the properties underlying the impact of Nrxn overexpression on inhibitory synaptic transmission are largely unknown, the Nrxn-GABA_A_R is interaction is cell autonomous and independent of Nlgn2 binding. The mapping of the precise interaction interface and whether this interaction occurs in *cis* or *trans* remains to be tested.

### IgSF21

Immunoglobulin superfamily member 21 (IgSF21) was recently identified as a Nrxn2α-specific interacting protein (Tanabe et al., 2017). IgSF21 contains two immunoglobulin domains (Ig1 and Ig2) and is anchored to the post-synaptic membrane *via* a GPI modification. Cellular binding assays revealed that the interaction between IgSF21 and Nrxn2α required Ig1 of IgSF21 and LNS1 of Nrxn2α (Tanabe et al., 2017). IgSF21 gene is expressed at both the embryonic and postnatal stages and its protein is highly expressed in cortex, hippocampus, thalamus, and pons. Constitutive deletion of IgSF21 reduced the levels of inhibitory synaptic proteins, reduced inhibitory synapse densities and functionally impaired inhibitory synaptic strength. Whether the phenotypes observed in the IgSF21 KO are a consequence of developmental or postnatal loss of *trans*-synaptic signaling with Nrxn2α or other interacting proteins is unknown.

## Conclusion

The expression and function of neurexins and their ligands at inhibitory synapses is an emerging area of study, having been historically overlooked in favor of investigation of excitatory synapses. It is clear from several decades of work that neurexins specify multiple key aspects of neurotransmission at glutamatergic synapses. Accumulating evidence indicates these synaptic adhesion molecules play critical roles at inhibitory synapses as well by mediating pre- and post-synaptic properties of synaptic transmission and maintaining connectivity. A significant challenge to understanding the functional role of neurexins at inhibitory synapses has been accounting for the profound diversity of inhibitory neuron classes in the brain. Thus far, many studies have focused on defining Nrxn and Nrxn-ligand function in the three prominent inhibitory classes in cortex and hippocampus, PV, SOM, and CCK GABAergic interneurons. Together these studies demonstrate that neurexin expression and function is cell-type and region-specific. This has been most extensively demonstrated in studies of Nrxn3, which mediates different synaptic properties depending on the brain region, identity of the pre- and post-synaptic neuron, and sex of the animal.

Due to current technical limitations, one major difficulty has been determining the precise mechanisms by which these diverse functions are manifested, as cell-type specific differences in neurexin isoform and alternative splicing mRNA expression profiles do not fully explain all cases of functional diversity. Heterogeneity could also be mediated by synapse-specific expression or localization of neurexin proteins as well as synapse-specific signaling, both intracellularly and *trans*-synaptically *via* neurexin ligands. Indeed, as we discuss here, Nrxn ligands also exhibit brain-region and synapse-specific localization and function at inhibitory synapses. Thus, in addition to continuing to explore individual neurexin function in other classes of inhibitory neurons and in other brain regions, future studies should also aim to clarify these possible mechanisms as new tools to interrogate Nrxn localization become available. Finally, a major gap in the literature is whether neurexins control synaptic plasticity at inhibitory synapses, like they do at excitatory synapses. Similarly, several studies have revealed dynamic, circuit-specific changes in individual neurexin expression following behavior and experience, but it is largely unknown if these changes are occurring in inhibitory neurons, and if they are, how this may be associated with inhibitory synaptic plasticity. Neurexins are consistently implicated in several neuropsychiatric and developmental disorders that are thought to be in part driven by deficits in inhibitory function and dysregulation of E/I balance, such as ASDs, schizophrenia, epilepsy, substance use disorders, and stress. Continued investigation of the expression, localization, and function of neurexins in inhibitory transmission at a synapse-specific and activity-dependent level will be crucial to elucidate how these molecules contribute to brain function in both healthy and disease states.

## Author contributions

EB and JA wrote the article. Both authors contributed to the article and approved the submitted version.
